# *In Vitro* Selection of Fab Fragments by mRNA Display and Gene-Linking Emulsion PCR

**DOI:** 10.1155/2012/371379

**Published:** 2012-09-23

**Authors:** Takeshi Sumida, Hiroshi Yanagawa, Nobuhide Doi

**Affiliations:** Department of Biosciences and Informatics, Keio University, 3-14-1 Hiyoshi, Kohoku-ku, Yokohama 223-8522, Japan

## Abstract

*In vitro* selection by display methods has been an effective tool for engineering recombinant antibodies. mRNA display based on a cell-free translation system has the advantages of larger library sizes and quicker selection procedures compared with cell-based display methods such as phage display. However, mRNA display has been limited to select single-chain polypeptides such as scFvs due to its characteristic of linking a nascent polypeptide with its encoding mRNA on the ribosome. Here we demonstrated a new way of selecting heterodimeric Fab fragments by using mRNA display combined with emulsion PCR. We designed a pair of complementary 5′ UTR sequences that can link the Fab heavy and light chain genes together by overlap-extension PCR in water-in-oil emulsions. We confirmed that two mRNA-displayed polypeptides for heavy and light chain of a model Fab fragment were associated into the active form and that a specific Fab fragment gene was enriched over 100-fold per round of a model affinity selection followed by the gene-linking emulsion PCR. We further performed directed evolution of Fab fragments with higher binding activity from a randomized Fab fragment library.

## 1. Introduction

*In vitro* selection by display methods has been an effective tool in the field of protein engineering and especially has been used to engineer recombinant antibodies for various biological applications [1]. Phage display has been widely used in the industry due to its feasibility to select Fab fragments [2]. The Fab fragment of an immunoglobulin is a heterodimer of the N-terminal half of a heavy (H) chain and a complete light (L) chain. Because the Fab is more native-like than the single-chain Fv (scFv), which is the other commonly used recombinant antibody format for *in vitro* selection, the Fab fragment format makes it able to select more practical antibodies [3]. Other than phage display, cell-free translation-based methods such as ribosome display [4] and mRNA display [5] are being used for *in vitro* selection of antibodies due to its advantage of permitting speedier selection from larger size libraries than cell-based methods. However, these cell-free translation-based methods are limited to select scFvs due to its characteristic of linking a nascent polypeptide with its encoding mRNA on the ribosome.

 To overcome this limit, we have recently developed a bicistronic DNA display to select Fab fragments in a cell-free translation system [6]. Bicistronic DNA display relies on *in vitro* compartmentalization in water-in-oil emulsions [7], and the man-made cell-like compartments make it possible to display oligomeric proteins in a cell-free translation system. Although bicistronic DNA display has made it possible to select Fab fragments in a cell-free translation system, it has some disadvantages compared with mRNA display. First, the initial library size of bicistronic DNA display is three orders of magnitude less than that of mRNA display. Second, the linkage between the DNA and protein is a streptavidin-biotin complex, making it less stable compared with the covalent bond in mRNA display.

 In this study we combined emulsion PCR [8–11] with mRNA display in order to be able to select Fab fragments by mRNA display. Since mRNA display is capable of selecting candidates from a more diverse library and designing a more flexible selection strategy compared with bicistronic DNA display, this new method would provide a new option for selecting Fab fragments in a cell-free translation system.

## 2. Results and Discussion

### 2.1. Strategy

**Figure 1:**
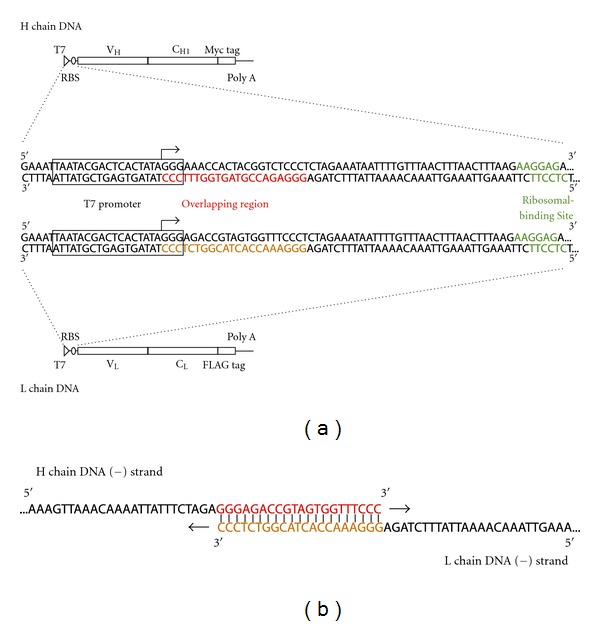
The DNA construct of the Fab fragments for mRNA display. (a) From the 5′ end it consists of a T7 promoter (T7), ribosomal-binding site (RBS), variable region and constant region of the H chain or L chain, epitope tag, and a poly A tail. The H chain epitope tag is a Myc tag and the L chain is a FLAG tag. The poly A tail is for purification of mRNA-displayed molecules by biotin-oligo dT in combination with streptavidin beads. (Middle) Details of the linkable 5′ UTR. Between the T7 promoter (boxed) and RBS (green), 21 bases from the beginning of T7 promoter transcription start point (arrow), in other words +1 to +21 of the H chain DNA (red) and L chain DNA (orange) are designed so that they overlap with each other during overlap-extension PCR. (b) The reverse-transcribed DNA strands overlap at the overlapping region (red) to form an H chain gene and L-chain-gene-linked DNA.

A Fab fragment consists of an H chain and an L chain, and by applying mRNA display, an mRNA-displayed H chain and an mRNA-displayed L chain can each be made. If these two mRNA-displayed molecules dimerize, they will form an mRNA-displayed Fab fragment. However, in this case, the correspondence of the selected H and L chains cannot be determined because the two genes are different RNA molecules and will be amplified separately after affinity selection. Applying overlap-extension PCR in water-in-oil emulsion from a single Fab molecule and linking these two genes together to amplify them at once will overcome this problem. Thus, we have designed a pair of complementary 5′ UTR sequences that can be linked together by overlap-extension PCR (Figure 1). The whole DNA construct for this strategy consists of a linkable 5′ UTR with a T7 promoter and ribosomal binding site; an ORF with the variable region, constant region, and an affinity tag, and at the 3′ end there are 25 adenines for mRNA-based purification by oligo-dT resin.

**Figure 2:**
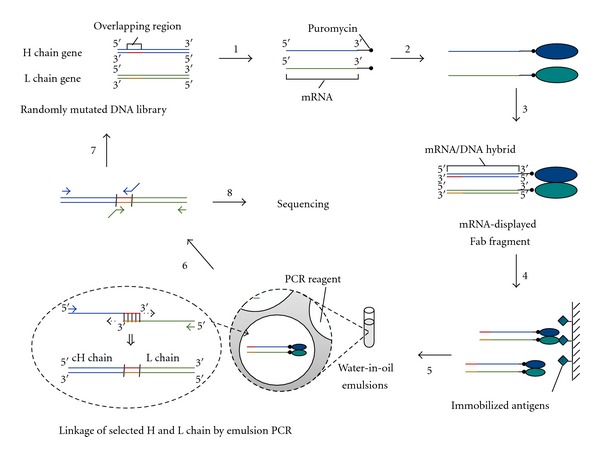
Scheme of *in vitro* selection of Fab fragments using mRNA display and emulsion PCR. Step 1: A randomly mutated DNA library of an H chain gene and an L chain gene is separately prepared. Each DNA library is transcribed and puromycin is ligated to the 3′ end to make an mRNA template. Step 2: The mRNA template is translated to form an mRNA-displayed molecule. Step 3: The molecules are purified and subsequently reverse is transcribed to make the mRNA portion a DNA hybrid. The H and L chain molecules are combined to form an mRNA-displayed Fab fragment. Step 4: The mRNA-displayed Fab fragments are subjected to *in vitro* antigen selection. Step 5: The selected mRNA-displayed Fab fragments are recovered and mixed with PCR reagents. The mixture is emulsified and the corresponding H and L chain genes are linked together by overlap extension PCR. Step 6: The linked DNA is amplified by PCR again with different primers to regenerate the H and L chain genes. Step 7: The regenerated genes are reamplified by DNA shuffling to make the templates for the next round of selection. Step 8: After a suitable number of rounds of selection, the selected DNA is cloned and sequenced to identify the selected Fab fragments.

 The scheme for *in vitro* selection of Fab fragments using mRNA display and emulsion PCR is shown in Figure 2. Firstly, mRNA-displayed H and L chains are separately prepared by *in vitro* translation of puromycin-ligated mRNA templates. Both the H chain and L chain are subsequently purified by oligo-dT resin in order to remove all free proteins that could not form an mRNA-displayed molecule, avoiding a Fab fragment existing with only one chain being mRNA-displayed. After a reverse transcription step in order to make the mRNA portion of the mRNA-displayed molecule an mRNA/DNA hybrid, the H and L chains are mixed together to form mRNA-displayed Fab fragments. These mRNA-displayed Fab fragments are selected by the target antigen and then eluted under conditions that the oligomeric structure is maintained. The eluted mRNA-displayed Fab fragments are subjected to emulsion PCR and a single mRNA-displayed Fab fragment is trapped inside a single micelle where the H and L chain genes are linked together by overlap-extension PCR. The linked DNA is either amplified by PCR to regenerate the H and L chain genes for further selection or sequenced to identify the selected Fab fragments.

### 2.2. Proof-of-Principle Experiments

 In order for the scheme depicted in Figure 2 to work, we confirmed the following three points: (i) the H and L chain genes overlap properly by emulsion PCR; (ii) an mRNA-displayed H chain and an mRNA-displayed L chain form a Fab fragment with binding activity; (iii) genes do not crossover during emulsion PCR and the corresponding H chain and L chain genes are properly linked together.

**Figure 3:**
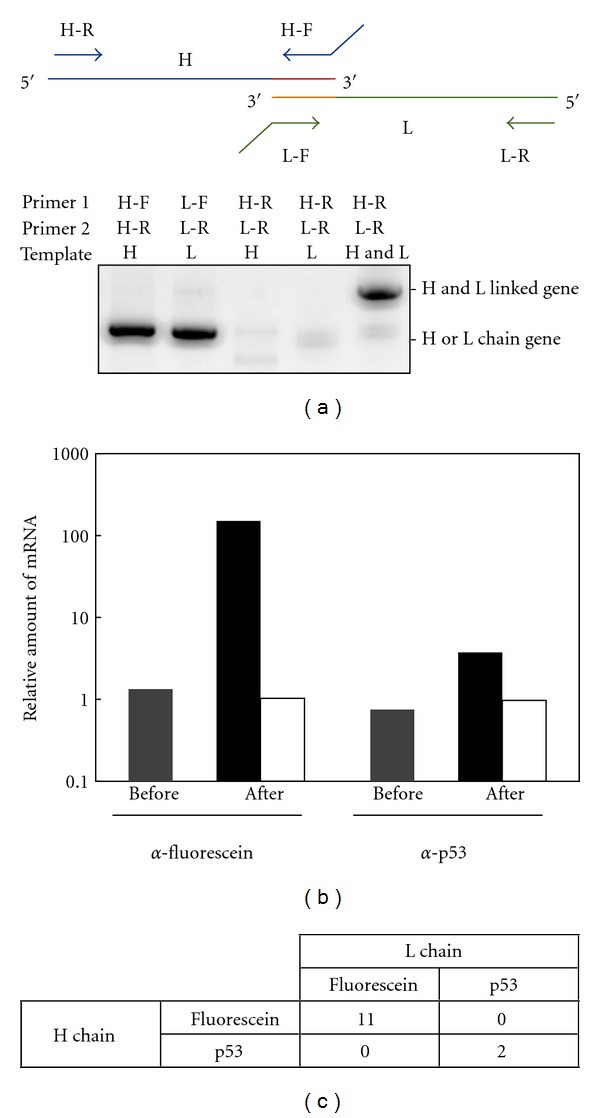
Model experiments of *in vitro* selection of Fab fragments using mRNA display and emulsion PCR. (a) Confirmation of the H and L chain gene linking by PCR. The H chain or/and L chain gene of anti-fluorescein Fab fragments were amplified by PCR with forward and reverse primers of the corresponding gene or only reverse primers of both genes and analyzed on a 1.5% agarose gel. Primers H-F, H-R, L-F and L-R are UnivOL1-F, Myc-R, UnivOL2-F, and FLAG-R, respectively (Table 1). (b) Antigen-specific enrichment of mRNA-displayed Fab fragments. A single round of affinity selection was carried out for antigen-immobilized and nonimmobilized beads from a mixture of equally amounted anti-p53 and anti-fluorescein Fab fragment genes. The amount of each gene before and after selection was quantified by quantitative PCR. The amount of the positive control gene divided by that of the negative control gene is plotted for both before selection (gray) and after selection by antigen-immobilized bead (black) and nonimmobilized beads (white). (c) Confirmation of H and L chain gene linking by cloning and sequencing after affinity selection. mRNA-displayed anti-fluorescein Fab fragments and mRNA-displayed anti-p53 Fab fragments were mixed in a ratio of 1 : 50 and then used for affinity selection against fluorescein-immobilized beads. After selection, a total of 13 clones were sequenced, and the correspondence of the H and L chains was confirmed.

Figure 3(a) shows that when there are only reverse primers for the H and L chains, the DNA amplifies only when both the H and L chain genes exist and does not when there is only one of the genes. This proves that the linkable 5′ UTR is properly designed to overlap and only Fab fragments with both mRNA-displayed H and L chains can be specifically amplified. Therefore, unwanted gene amplification of unspecific H chain or L chain binders that does not form Fab fragments can be removed, exhibiting the advantage of this overlapping method compared to regular PCR amplification.

 Next, to show that an mRNA-displayed H chain and mRNA-displayed L chain form a Fab fragment with binding activity, mRNA-displayed anti-fluorescein Fab and anti-p53 Fab fragments were used in a model affinity selection experiment. Confirmed by pull-down assays and western blotting, these antibodies do not bind unless they form a Fab fragment (data not shown). Equal molar amounts of mRNA-displayed anti-fluorescein Fab and anti-p53 Fab fragments were subjected to *in vitro* selection against antigen p53 or fluorescein, and the amounts of the molecules before and after selection were quantified by quantitative PCR. As expected, each of the Fab fragment genes was enriched when they were selected against their antigens (Figure 3(b)). The anti-fluorescein Fab fragment gene showed an approximate 110-fold enrichment and the anti-p53 Fab fragment gene showed an approximate 5-fold enrichment. The difference in the enrichment efficiency is probably due to the difference in the dissociation constant; the anti-p53 Fab fragment has a weaker affinity towards its antigen. On the other hand, both antibodies did not show any enrichment when their antigens were not present during *in vitro* selection, showing that both mRNA-displayed Fab fragments bind to their antigens with specificity.

 Finally, to confirm that genes do not crossover during emulsion PCR and the corresponding H chain and L chain genes are properly linked together, the DNA after affinity selection was cloned and confirmed by capillary DNA sequencing. The mRNA-displayed anti-fluorescein Fab fragment and the mRNA-displayed anti-p53 Fab fragment were mixed in a ratio of 1 : 50 and then used for affinity selection against fluorescein-immobilized beads. Out of the 13 sequenced DNA clones after selection, 11 clones were H and L chain-linked anti-fluorescein and 2 clones were H and L chain-linked anti-p53 (*P* < 0.001), resulting in a conclusion that the corresponding H chain and L chain genes are properly linked together and the genes do not crossover during emulsion PCR (Figure 3(c)).

### 2.3. Affinity Selection from a Randomized Fab Fragment Library

**Figure 4:**
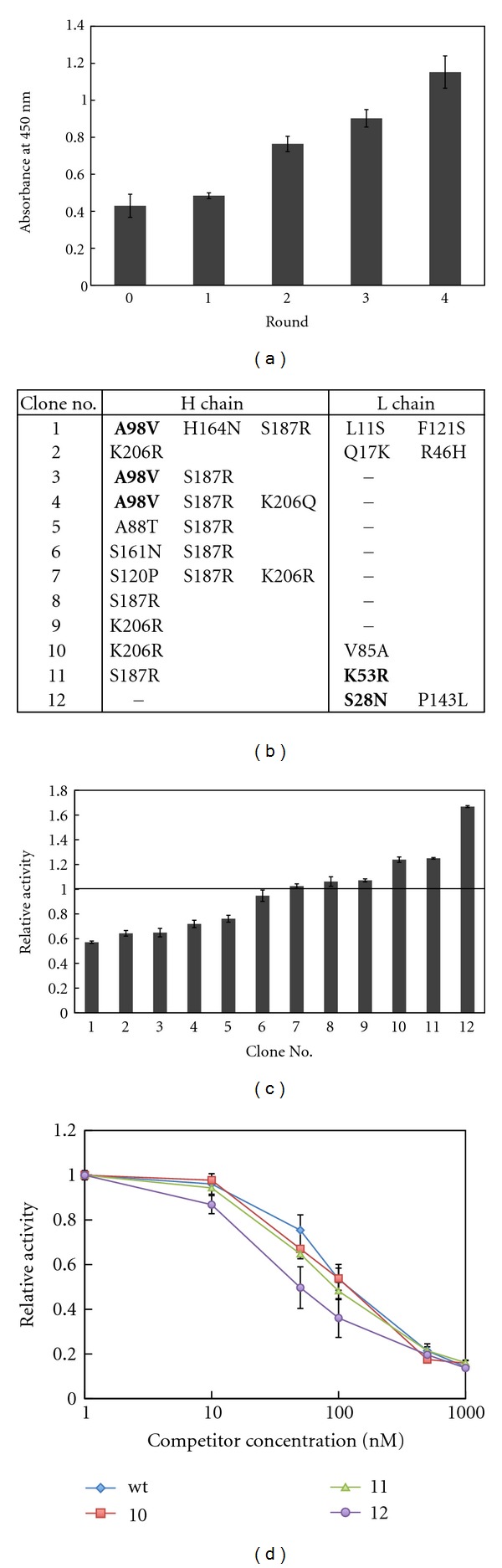
Affinity selection of randomly mutated anti-p53 Fab fragments. (a) Binding activity of the library after each round of selection. Random mutations were introduced to anti-p53 Fab fragments and 4 rounds of affinity selection were carried out under decreasing antigen concentration (round 1, 400 nM; round 2, 40 nM; round 3, 4 nM; round 4, 0.4 nM). After each round of selection, the fraction of the mutant Fab fragment library that binds to p53 was monitored by ELISA. Round 0 represents the randomly mutated initial library before selection. (b) Amino acid mutations of the selected variants. Bold type represents the mutations in the CDR and the minus (−) represents no amino acid mutations. (c) Binding activity of the selected variants after the 4th round of selection. The antigen binding activities of the selected variants were measured by ELISA. The absorbance at 450 nm of the wild type was used for normalization. (d) Competitive ELISA for estimating their affinities of variants with higher binding activity than the wild type. Competitive ELISA was preformed with 0–1000 nM free p53 for clone number 10–12 and the wild type (wt). The signals were normalized to that of 0 nM competitor concentration.

Finally, we applied the mRNA display and emulsion PCR procedure for selection from a randomized Fab fragment library. We constructed an anti-p53 Fab fragment library with random point mutations by error-prone PCR and DNA shuffling from the wild type. When a fraction of the library was analyzed by DNA sequencing, the H chain and L chain had an average of 2.9 base/gene and 3.1 base/gene mutation, respectively. From this library, 4 rounds of affinity selection were performed under the condition of gradually decreasing amounts of immobilized antigen (round 1, 400 nM; round 2, 40 nM; round 3, 4 nM; round 4, 0.4 nM). After 4 rounds of selection, the total binding activity of *in*-*vitro*-translated products of the library at each round was analyzed by ELISA (Figure 4(a)). The binding activity gradually increased in successive rounds of selection, indicating that specific binders have been enriched in the library.

 The library after the 4th round of selection was cloned and sequenced (Figure 4(b)). The binding activities of arbitrarily chosen 12 clones were analyzed and approximately 60% of those clones had similar or higher binding activities than the wild type (Figure 4(c)). The clones that had similar binding activities as the wild type (numbers 6–9) had mutations only in the constant region and since the constant region does not influence the affinity of most antibodies [3, 6] it is likely that these are neutral mutations. Among these mutations, S187R and K206R were found in several other clones and, these may be fixed neutral mutations in an early round of selection by random genetic drift. The clones that had higher binding activity than the wild type (numbers 10–12) all had mutations in the variable region of the L chain, either in the CDR or close to the CDR. Since the CDR directly contacts with the antigen, mutations in and near the CDR affect the affinity of the antibody [5, 12–14]. Analysis by competitive ELISA confirmed that all these clones specifically competed against free p53 (Figure 4(d)), and from the Scatchard plot, the *K*_*d*_ of clone 10, 11, 12 and wild type were estimated to be around 100 nM, 90 nM, 60 nM, and 140 nM, respectively. 

 These results demonstrated that the combination of mRNA display and emulsion PCR was able to eliminate inactive Fab fragments from the randomized Fab fragment library and select Fab fragment candidates according to the designed procedure. However, the affinities of the selected mutants were not so high. Only mutations in the L chain had a positive influence on the affinity, and mutations in the H chain were either neutral (S187R, K206R) or slightly harmful (A98V). Mutants with A98V still have intermediate binding activity and the rather mild selection pressure of decreasing antigen concentration in this study may have let such a mutation survive through selection. Gradually lowering the antigen concentration has been demonstrated as a strategy for affinity maturation of some antibodies [15, 16] but introducing more stringent selection pressure such as off-rate selection [5, 12–14] may have produced a better result. Further, the difference in the cell-free translation system may have affected the result as well. In this study, a reconstituted PURE *E. coli* translation system [17] was used for mRNA display of antibody fragments for the first time, but the efficiency of the mRNA being linked to the protein (~10% of the total mRNA library; data not shown) is lower than our previous study based on a wheat germ translation system [5]. Optimizing the cell-free translation system for our mRNA display system should produce better results as well.

### 2.4. Future Perspectives

A big advantage of our method in this study may be the large library size of mRNA display, which is the largest among all display methods, and the use of next generation sequencers would be able to pull out the full potential of this method. The newest Roche 454 Sequencer can sequence approximately 10^6^ reads of approximately 900 bp, long enough to cover both of the linked variable regions. Also, a recent study by phage display and deep sequencing has revealed that one round of selection is enough for identifying positive clones [18]. Although, 10^6^ reads are not enough to cover the whole selected Fab fragment library, combining it with a microfluidic chip for high enrichment efficiency per round of selection [19] may make it possible to obtain unique high affinity binders. Further possibilities may be considered when the specifications of the next generation sequencers improve even more. The speed of improvement for this technology is remarkable, and when it becomes possible to sequence the whole selected Fab fragment library (around 10^8^–10^9^ molecules), it should allow selection of low affinity antibodies that would usually be lost in a typical selection of repetitious rounds, expanding the variety of potentially effective antibody candidates.

 Other possible applications by our method described in this study would be proteome analyses, such as massively parallel detection of protein/protein interactions. Recently, Nirantar and Ghadessy demonstrated a way to identify various protein/protein interactions by library versus library two-hybrid screening using emulsion PCR [20]. A similar strategy can be carried out by our method by pulling-down *in*-*vitro*-translated mRNA-displayed protein complexes with an affinity tag and incorporating them into emulsions. Furthermore, the immense flexibility of our cell-free translation-based method would allow selection of not only protein/protein complexes, but also RNA/protein complexes. This concept can be completed by simply changing one side of the complex from an mRNA-displayed molecule to an ordinary RNA molecule, in other words merging SELEX [21], a common way for *in vitro* selection of RNA aptamers, with this mRNA display and emulsion PCR method. An example of RNA/protein complex selection would be selection of a ribonucleic peptide aptamer. In previous studies, it took two steps to make a high-affinity ribonucleic peptide aptamer against ATP, first step by SELEX [22] and second step by phage display [23]. Since our method can do selection against RNA and peptide at once, it may be possible to obtain a high-affinity binder in only one step. We have confirmed that emulsion RT-PCR can be carried out and incorporated into this method (data not shown), and selection of RNA/protein complexes by *in vitro* display methods shall be carried out in the near future.

 In conclusion, other than *in vitro* selection of Fab fragments, our method in this study has the possibilities of being able to carry out proteomics applications and RNA/protein complex selection. This variety of possible applications shows the potential convenience of *in vitro* selection by mRNA display and gene-linking emulsion PCR.

## 3. Materials and Methods

### 3.1. DNA Construction

The oligonucleotide sequences used in this study are listed on Table 1. The H chain and L chain genes of both anti-fluorescein Fab and anti-p53 Fab fragments were constructed by PCR with KOD-plus Neo DNA polymerase (Toyobo) from plasmids including these Fab fragment genes [6] using primers Universal-OL1 and Myc-R or Universal-OL2 and FLAG-R, respectively. The PCR products were cloned into pCR2.1-TOPO vector (Invitrogen) and confirmed by an ABI PRISM 3100 genetic analyzer (Applied Biosystems). DNA templates of the H chain and L chain genes for mRNA display were prepared by PCR from these plasmids using primers Universal-OL1 and Myc25A-R, or Universal-OL2 and FLAG25A-R, respectively.

### 3.2. Library Construction

A randomized anti-p53 Fab fragment library was constructed by introducing point mutation to the wild type with the combination of error-prone PCR and StEP [24]. Mutazyme II DNA polymerase (Stratagene) was used for error-prone PCR and 15 ng of either the H chain gene or the L chain gene constructed above was amplified for 30 cycles according to the protocol. After error-prone PCR, StEP was performed using *Ex Taq* DNA polymerase (Takara) as follows: denaturation at 95°C for 2 min, 80 cycles of 95°C for 30 sec and 55°C for 5 sec, followed by 95°C for 30 sec, 60°C for 30 sec, and 72°C for 15 min. The PCR products were then resolved by agarose gel electrophoresis, extracted and purified with the QIAquick gel extraction kit (Qiagen). Error-prone PCR and StEP was performed once more each under the same condition. At every step, primers Universal-OL1 and Myc25A-R or Universal-OL2 and FLAG25A-R were used for the H chain and L chain genes, respectively.

### 3.3. In Vitro Transcription and Translation

The DNA templates were transcribed by T7 Ribomax Express Large Scale RNA production system (Promega) and purified by using the RNeasy mini kit (Qiagen). The transcribed mRNAs were then ligated with a puromycin linker by T4 RNA ligase (Takara) as described in previous studies [5]. Translation was done by PURE system S-S (PostGenome Institute) and 5 pmol each of H and L chain templates were translated separately in a 25 *μ*L scale for 2 hours at 37°C to form mRNA-displayed molecules. The molecules were then diluted into 175 *μ*L of Hybridization Buffer (1 M NaCl, 100 mM Tris-HCl, pH 7.4, 10 mM EDTA, and 0.25% Triton X-100) and mixed with 100 pmol of poly-dT oligonucleotide with a biotinylated photo-cleavable linker and a high-capacity NeutrAvidin agarose resin (Pierce). The resin mixture was mixed gently at 4°C for 1 hour and subsequently washed with PBST (PBS with 0.1% Tween 20) three times. The resin was then mixed with ReverTra Ace reverse transcriptase (Toyobo) and incubated at 42°C for 30 min. After washing with PBST three times, 40 *μ*L of elution buffer (PBS with 10% Solution A of PURE system S-S) was added to the resin and exposed to UV radiation at >300 nm to elute the mRNA-displayed molecules as previously described [25]. The purified mRNA-displayed H and L chains were mixed and then incubated overnight at 4°C to form mRNA-displayed Fab fragments.

### 3.4. Gene-linking Emulsion PCR

Emulsions were prepared by stirring 50 *μ*L of PCR reagents containing the selected mRNA-displayed Fab fragments, KOD-plus Neo DNA polymerase, and primers Myc-R and FLAG-R into 950 *μ*L of mineral oil-surfactant mixture [mineral-oil (Nacalai Tesque) containing 0.45% Span 85 (Nacalai Tesque), 0.04% Tween 20 (Sigma), and 0.01% Triton X-100 (Nacalai Tesque)] at 2300 rpm for 30 sec at 4°C. The emulsions were dispensed into 0.2 ml PCR tubes 80-*μ*L each and PCR was performed with a T1 thermocycler (Biometra). The PCR program was as follows: denaturation at 94°C for 2 min; 40–50 cycles of 98°C for 10 sec, 60°C for 30 sec, and 68°C for 1 min; final extension at 68°C for 4 min. A 70 *μ*L aliquot of the top layer was collected from each PCR tube, moved to a 1.7 mL tube, mixed with 100 *μ*L of ddH_2_O, and centrifuged at 15,000 rpm for 10 min at 40°C to break the emulsions. Approximately 90% of the aqueous layer from the bottom was recovered and purified by briefly mixing it with 1 mL of mineral oil and centrifuging the mixture at 15,000 rpm for 2 min. From the purified aqueous layer, a 120 *μ*L aliquot was recovered and purified by using the QIAquick PCR purification kit (Qiagen). Subsequently, the PCR products were resolved by agarose gel electrophoresis, extracted, and purified with the QIAquick gel extraction kit.

### 3.5. Affinity Selection

Biotinylated antigen (either fluorescein (Sigma) or p53 C-terminal peptide (SKKGQSYSRH)) or biotin was added to 100 *μ*L of Magnotex-SA beads (Takara) dispersed in PBST, gently mixed at 4°C for 1 hour. After washing with PBST three times, 100 *μ*L of blocking buffer (DIG wash and block buffer set; Roche) was added and gently mixed at 4°C for another hour and finally washed with PBST to prepare antigen-immobilized beads or mock beads, respectively. To these antigen-immobilized beads or mock beads 5 *μ*L of sonicated salmon sperm DNA (Stratagene), 5 *μ*L of yeast tRNA (Invitrogen) and 40 *μ*L mRNA-displayed Fab fragments were added. After having been gently mixed for 1 hr at 4°C for binding, the beads were washed with PBST three times and heated at 70°C for 15 min to elute the bound mRNA-displayed Fab fragments. Subsequently, emulsion PCR was performed as described above to link the corresponding H and L chain genes together.

 To regenerate the genes, PCR was performed with KOD-plus Neo DNA polymerase using primers UnivOL1-F and Myc-R or UnivOL2-F and FLAG-R for the H chain and L chain genes, respectively. To prepare the DNA templates for the next round of selection, the regenerated H chain and L chain genes were amplified by StEP using *Ex Taq* DNA polymerase and the same primers and program described above with exception that the cycle numbers ranged from 60–80 cycles. The PCR products were then resolved by agarose gel electrophoresis, extracted and purified with the QIAquick gel extraction kit.

 After several rounds of selection, the selected DNA was cloned using a TOPO XL PCR cloning kit (Invitrogen) and sequenced with the ABI PRISM 3100 genetic analyzer.

### 3.6. Quantitative PCR

The DNA amount of each gene was quantified by real-time PCR using SYBR premix *Ex Taq* DNA polymerase (Takara) and Lightcycler (Roche). Primers Fluo-F and Fluo-R were used for anti-fluorescein Fab fragment genes, and primers p53-F and p53-R were used for anti-p53 Fab fragment genes.

### 3.7. ELISA

Fab fragments were expressed by transcribing and translating the H chain and L chain gene DNA with the PURE system S-S. Meanwhile, a p53 C-terminal peptide-immobilized plate was prepared by adding 100 pmol of biotinylated p53 C-terminal peptide and 100 *μ*L of TBST (TBS with 0.1% Tween 20) to a Streptavidin C8 transparent plate (Nunc) and shaking it for 1 hour at room temperature. The plate was then washed with TBST 10 times and blocked with 200 *μ*L of blocking buffer by shaking it for 1 hour at room temperature. Separately, the expressed Fab fragments were diluted into 100 *μ*L of TBST containing 0–1000 nM p53 C-terminal peptide as a competitor and preincubated at 4°C for 1 hour. Then, the samples were added to the fluorescein-immobilized plate and shaken for 5 min. After a washing step, 100 *μ*l of TBST with 0.1% anti-FLAG M2 peroxidase conjugate (Sigma) was added and shaking was continued for 1 hour. The plate was washed for the last time and 100 *μ*L of TMB (Nacalai Tesque) was added. The plate was shaken for 20 min and then 100 *μ*L of 1 N H_2_SO_4_ was added to stop the reaction. The absorbance at 450 nm was measured (reference wavelength: 655 nm). The *K*_*d*_ values of the selected clones were estimated from the Scatchard plot.

## Figures and Tables

**Table 1:** Oligonucleotide sequences.

Name	Sequence (5′ to 3′)
Universal-OL1	GAAATTAATACGACTCACTATA**GGGAAACCACTACGGTCTCCC**TCTAGAAATAATTTTGTTTAACTTTAAGAAG GAGATATACCA
Myc-R	AAGGTCTTCTTCACTAATCAGTTTCTGCTC
Universal-OL2	GAAATTAATACGACTCACTATA**GGGAGACCGTAGTGGTTTCCC**TCTAGAAATAATTTTGTTTAACTTTAAGAAG GAGATATACCA
FLAG-R	CTTGTCGTCATCGTCCTTGTAGTC
Myc25A-R	TTTTTTTTTTTTTTTTTTTTTTTTTAAGGTCTTCTTCACTAATCAGTTTCTGCTC
FLAG25A-R	TTTTTTTTTTTTTTTTTTTTTTTTTCTTGTCGTCATCGTCCTTGTAGTC
PolyT oligo	TTTTTTTTTTTTTTTTTTTTTTTTT
UnivOL1-F	GAAATTAATACGACTCACTATAGGGAAACCACTACGGTC
UnivOL2-F	GAAATTAATACGACTCACTATAGGGAGACCGTAGTGGTT
Fluo-F	CAGGATCGAGTGGGTCAAAC
Fluo-R	GGTGCTGGAGCTTTTGTCTG
p53-F	GGCAGAGCTTGTAAGGTCAG
p53-R	CAATCCATCCAATCCACTCC

The bold type resembles the sequences of the overlapping region for overlap-extension PCR.

Poly T oligo is labeled with a photo-cleavable biotin at the 5′ end.
